# The Organization of International Dental Congresses

**Published:** 1903-02

**Authors:** 


### THE ORGANIZATION OF INTERNATIONAL DENTAL CONGRESSES.

To many of our readers the continuation of the controversy in regard to the Fourth International Dental Congress may seem an unprofitable waste of time, and it would be were it not for the fact that the real issue is not personal, but involves not only this congress, but any others that may be held in the future.

The question that requires an answer is, Have these congresses, from that held in Paris in 1889 to the one proposed for 1904 at St. Louis, been a continuous series and regulated by a resolution or resolutions passed by the original congress? It is presumed no one will attempt to answer this affirmatively, for there is, apparently, no evidence that any attempt was made at the Congress in 1889 to regulate any succeeding one. It was regarded as strictly an international affair under national management, and any attempt to control other nationalities, in a similar effort, was evidently unthought of, and, if it had been undertaken, would doubtless have been resisted as an unwarranted interference with national rights.[1] When the "World's Columbian Dental Congress" in 1893

---

[1] The resolution adopted by the Congress of 1889 was as follows: "The vote of the Congress at its last session, September 6, 1889, declares that, under circumstances similar to those giving birth to the present congress, it is desirable that another congress be convened in the United States in 1893, at the time of holding the approaching World's Exposition." This is simply advisory, no attempt being made to enforce it. Dr.

was originally under consideration, there is no evidence to show that the previous congress was considered further than as an international organization, the good points of which might be worthy of adoption and the mistakes rejected.

In the *Dental Review,* of Chicago, for December, 1902, there is an exceedingly well-prepared paper on "Internationalism in Dentistry," by Dr. C. N. Johnson, in which only indirect allusion was made to international dental congresses, but confined the consideration of the subject to a temperate view of the international aspects of dentistry. This paper was, without apparent reason, made the text for a lengthened and evidently carefully prepared response on International Dental Congresses, by Dr. Harlan, of Chicago. This was placed in the discussion that followed the reading of the essay. It had been evidently prepared in advance to serve as an answer against the almost universal criticism made in this country in opposition to the action of the committee appointed by the Féderation Dentaire Internationale to look after the interests of the Fourth International Dental Congress. The active part that Dr. Harlan has taken in dental congress matters, both at home and abroad, entitles whatever he may have to say to respectful consideration. The question must, however, be decided upon the facts of history, and those obtainable by the present writer do not warrant the claims made by the committee appointed by the Féderation.

For a better comprehension of the subject reference must be made to the original organization of the World's Columbian Dental Congress. The following forms a part of the original resolutions appointing a committee to carry out the proposed second international congress:

"WHEREAS, It is desirable that any meeting then held should be at the instance of the American Dental Association and the Southern Dental Association, and organized by a joint committee by them appointed."

This was followed by a resolution appointing five members from each national organization, with power to add to its membership one, three, or five more members.

---

Harlan, in his address before the American Academy of Dental Science, on "The Evolution of the Dental Congress," November 16, 1892, says, "A resolution was offered on the last day of the assemblage (Congress, 1889), which did not make it obligatory on a committee to call another congress."

In the report of the World's Columbian Dental Congress, prepared mainly by Dr. A. W. Harlan, secretary-general, it is stated, "The World's Columbian Exposition organized the World's Congress Auxiliary. . . . The purpose of the World's Congress Auxiliary is to establish a series of congresses, in which the best workers in general science, philosophy, literature, art, agriculture, trade, and labor may meet to present their experiences and results obtained in all those various lines of thought up to the present time."

It is a well-understood fact in the history of this congress that there was a sharp conflict between Mr. Bonney, president of the Auxiliary, and the committee. Some were strongly opposed to the word congress, preferring to call it the "World's Columbian Dental Meeting," but Mr. Bonney settled this by insisting that it should be called a Congress, and it was so named.

It will be observed, from the foregoing, that while the Congress was originally called by the American and Southern Dental Associations, the real organizing power existed in the World's Congress Auxiliary, and that no intimation of any previous authority being given from the Congress of 1889 was mentioned, nor was the said congress considered in connection therewith.

This, the Second International Dental Congress, was held, and the entire dental world is familiar with the results. Its success warranted repetitions of similar organizations, to be held as circumstances might dictate. On page 209, Vol. I., of the Transactions of the Columbian Dental Congress will be found a resolution introduced by M. Chas. Godon, of Paris, in which he proposes a method by which future congresses may be held. The resolution was deemed of sufficient importance to warrant its presentation in the French, German, Spanish, and English languages. This resolution, covering as it does, the first attempt to manage dental congresses, is worthy of being repeated in full.

"The undersigned foreign delegates, considering the success achieved by the World's Columbian Dental Congress, as proved by the number of its members, and by the importance of its accomplishments, desiring to assure for the future the reunion of similar congresses, have the honor to present the following resolution:

"This Congress considers: 1. That under similar circumstances to those which led to the Congress of Paris in 1889 and the World's Columbian Dental Congress of Chicago in 1893, it could not be otherwise than advantageous to the profession to have similar International Dental Congresses in the future. 2. That it should be *delegated to the foreign dental societies to determine for their respective countries the time and place for the meeting of such future International Dental Congresses."* (Italics ours.)

This was signed by fifty-one members from foreign countries, ranging from France to Japan. In the discussion following the introduction of this resolution Dr. Cunningham, of England, emphasized it by saying that the organization of such congresses "should be left to the national dental societies of the respective countries to do their best to initiate such a movement." The resolution was unanimously adopted. There was no effort made to appoint any committee, nor was there *authority given anywhere,* in this voluminous report, for any set of officers to carry out the suggestions of the resolution. It would seem, in view of the fact that this congress had no power to pass a binding resolution such as this, that it would have been the duty of the president of the congress to have exercised his parliamentary privilege and ruled the resolution out of order.

The readers of this article should note the foregoing fact, for it bears directly on a statement made by Dr. Harlan where he says, "Finally, when they (the French dentists) wanted to hold a congress in Paris, what did they do? They wrote to the men who were connected with and closed up the World's Columbian Dental Congress, and *wanted to know if they could have authority to organize a congress in Paris. That authority was given, and they went to work and organized a congress* in Paris." (Italics ours.) Dr. Harlan's statement that such a request was made is without doubt correct, but what are we to think of the additional fact that *he* or *they* gave authority to hold this congress, an authority he or they did not possess? Who, it may be asked, were the persons who presumed to speak for the World's Columbian Dental Congress? When that Congress closed its sessions the only work left with the officers, and especially with the secretary-general and the treasurer, was to close out the report of the transactions and have the accounts audited. The Congress, and all connected with it, ceased to have further life or power. The resolution of Dr. Godon made no provision for any one to give consent to establish a congress in 1900, and the dentists of Paris could not have understood the situation when they made the request, for had they referred to the Godon resolution they would have found that it was left to "each country to determine the time and place of each congress;" and further, they then would have discovered that there was no committee or other body empowered to grant such a request. This brings the history up to the organization of the Third International Dental Congress of 1900.

It is unnecessary to consider the organization, or the great success of this congress; all that is a matter of historical record; but the resolutions passed at the closing of the Congress are all of them important, and several bear directly upon the organization of the proposed Fourth International Dental Congress of St. Louis.

"Resolution No. 11. That an International Federation shall be created." Adopted.

"Resolution No. 12. That the national committees that were organized for the Congress shall constitute the International Dental Federation." Adopted.

"Resolution No. 13. That at the last session of the Congress a committee of seven or nine members shall be nominated to consider the formation of the International Dental Federation, to propose members thereof for the approval of the national committees, and to prepare the next International Dental Congress."

"Resolution No. 14. The International Dental Federation shall be composed of all the national committees represented by an Executive Council.

"The first Executive Council, comprising seven or nine members, will be nominated by the members of the Third International Dental Congress, in its last general session, on Tuesday, August 14, and its *powers will expire at the opening of the Fourth International Dental Congress,* of the organization of which it shall have charge." (Italics ours.)

Resolution No. 15 concerns itself with the time of holding the Fourth International Dental Congress, "which shall be held (at the latest) in five years in the country which may seem best to the Executive Council."

There is some difference in the translations as given by Dr. Harlan and the report from which the writer quotes, but this is not sufficiently marked to require comment. In Resolution 14 he translates that the committee "is *charged to organize* the Fourth International Dental Congress," and the other is, "The organization of *which it shall have charge.*" There is quite a difference in the two renderings, although both give a certain power to the committee.

The question now resolves itself into, 1. Had the Third International Dental Congress, Paris, 1900, the power to confer on any subordinate organization the authority to take charge of future congresses? and 2. If this power was correctly conferred, had the Fédération Dentaire Internationale, the subordinate organization alluded to, authority to transfer that power to a committee to supersede that appointed by the dentists of the country proposing to hold the congress? It is very evident, from the wording of the resolution introduced by Dr. Godon in the World's Columbian Dental Congress, that no such power was given. Even admitting that said authority was transferrable from the Congress of 1893 to that of 1900, there was no body from the former congress to whom was given the duty to call a future international organization. Further, the delegates sent from various countries to the 1900 congress were not instructed to vote for a series of congresses; hence in so doing it was upon their individual responsibility and without the knowledge or authority given by their constituents. Those who joined the Congress without any special constituency certainly exceeded their limitations when they voted to bind other countries. The writer is not aware that this action of the Congress of 1900 has ever been confirmed by the dental organizations of the world, and until this is done it is not binding on any of the national organizations.

If it be accepted that the Congress of 1900 had the power to confer authority upon a subordinate organization to organize international dental congresses, then it must be conceded, upon a fair interpretation of the resolution, that the committee of nine has the power to assist in the organization of the Fourth International Dental Congress, but its *power ceases the moment the president, with his gavel, calls the first meeting to order, and all further management must be left in the hands of the committee appointed by the National Dental Association.* Inasmuch as this latter committee has the entire responsibility, it should have the most prominent place in all preliminary movements looking towards the organization of the Congress.

There is evidently a wide difference in the ruling of Dr. Godon, president of the Fédération Dentaire Internationale, in the two letters sent by him to this country. The quotation given in the

December number of this journal states distinctly that the committee of nine appointed by the Federation should "collaborate to the good organization of the Congress, and will place itself at the *disposition of the organizers.*" (Italics ours.) In the letter to Dr. Harlan, dated October 8, 1902, he states that this committee of nine "is commissioned to organize the Fourth International Dental Congress." He simply directs that the committee shall place itself "in communication with the president of the National Dental Association," but not a word about collaboration with the committee appointed by that body. He does write of the organizers of the Fourth Congress that they "ought to give to the committee, which we have named, the powers necessary to accomplish its mission." Exactly what Dr. Godon means by this must remain for future explanation. If, as he says, he must legally abide by the decision made at Stockholm, no other interpretation can be given to his ruling, but that the committee of the National Dental Association," but not a word about collaboration with the nine appointed in that city by the Fédération. If this is the correct view of his letter, and there is no appeal from this decision, then the Fourth International Congress will have died in its birth, as no American committee would for a moment entertain such a proposition. It is very evident, however, that the first letter, addressed as described, really contained what Dr. Godon considered the spirit of the original resolution,—that there must be a joint effort of both committees in furthering the great work.

If there is any reconsideration of this subject at Madrid in April, one fact should not be overlooked,—that the original invitation of the National Dental Association to the Fédération Dentaire Internationale to take part in the Fourth International Dental Congress is practically an invitation from the dentists of the United States to the dentists of Europe to join with them in this Fourth Dental Congress. To treat this as has been proposed means a discreditable and discourteous act. Will the Federation, at its meeting in Madrid, consent to occupy this position?

From the quotations made it must be evident to any unprejudiced reader that the Second International Dental Congress was simply a creation of the Columbian Exposition, and had no authority to repeat itself in any other country, and that the Third International Dental Congress exceeded its authority in attempting to make these congresses a permanent organization and subject to its ruling, and that the Fédération Dentaire Internationale has no power to force the National Dental Association of the United States to accept a committee appointed by that body. The entire subject needs careful revision and a foundation laid upon a legal basis that will free it from all criticism. In order that this may be accomplished, the writer would suggest that at the next meeting of the Federation, to be held in Madrid, Spain, in April, 1903, this body should request the committee of nine to withdraw all active efforts in the preliminary work of the Fourth Congress and occupy a subsidiary relation thereto. And further, he would suggest that the Fédération should request the committee of the National Dental Association to incorporate in its announcement of the Congress that an effort would be made at said Congress to have rules formulated for the formation and government of future congresses, and requesting dental societies throughout the world to properly instruct their delegates as to the course they would wish them to pursue. Until some such measure is adopted there will be constant dissatisfaction with dictatorial assumptions of unauthorized and necessarily illegal bodies.

For the committee of the Federation to attempt to assume control under present conditions would be not only undignified in character, but a subversion of international comity upon which all international congresses must be based. If this be not closely adhered to in their formation and work, these organizations will fail to carry out the original intent,—that of bringing together into more harmonious relations the diverse sentiments naturally developed by isolation and lack of that cosmopolitan spirit so essential to the formation of a truly international professional spirit.

In connection with the foregoing it is stated that the president of the St. Louis World's Exposition has announced the organization of World's Congresses to be held in St. Louis in 1904. Howard J. Rogers, chief of the Department of Education, is to be the Director of Congresses. The Advisory Board to work in conjunction with him will be as follows: Chairman, Nicholas Murry Butler, president of Columbia University; William R. Harper, president of the University of Chicago; R. H. Jesse, president of the University of Missouri; Henry S. Pritchett, president of the Massachusetts Institute of Technology; and Herbert R. Putnam, Librarian of Congress.

Power is given this board to determine the number and extent of the congresses, the special features of each, the prominent men to take part, the character of the programmes, and the methods of carrying out the enterprise. The idea is to give the series of congresses unity and connected purpose, and make their proceedings a valuable contribution to the world's literature.

It would seem from this that upon the consent of this board will depend whether the International Dental Congress will have a place at the exposition of 1904.

